# The continuum of simulator-based maritime training and education

**DOI:** 10.1007/s13437-021-00242-2

**Published:** 2021-06-07

**Authors:** Tae-eun Kim, Amit Sharma, Morten Bustgaard, William C. Gyldensten, Ole Kristian Nymoen, Hasan Mahbub Tusher, Salman Nazir

**Affiliations:** Department of Maritime Operations, Faculty of Technology, Natural Sciences and Maritime Sciences, University of South-Eastern Norway, Postboks 4 3199, Borre, Norway

**Keywords:** Maritime education and training, COVID-19, Simulator, Assessment, Cloud-based simulation, Remote simulation

## Abstract

The COVID-19 pandemic has brought unprecedented challenges to the maritime supply chain and called for accelerated adoption of digital technologies in various aspects of maritime operations, including the area of maritime education and training (MET). This paper aims to discuss the current maritime simulator-based training and educational practices that forms an integral part in seafarer training and competency development. The study provides a review of the existing simulators in use in MET, and discusses upon the technological and pedagogical advancement of maritime simulator-based training interventions with predictions regarding the future MET practices with use of virtual reality and cloud-based simulators. This study—by focusing on ship’s bridge operations—highlights the characteristics of various types of simulators and also discusses the role of instructors, challenges, and opportunities involving future simulator-based MET due to accelerated adoption of digital technologies and the need to comply with pandemic-related restrictions for MET institutes. The analysis generated in the paper may contribute to the ongoing discussion regarding the future of simulator-based MET and the fulfillment of the UN Sustainable Development Goal (SDG) 4 in the maritime sector.

## Introduction

The ongoing COVID-19 pandemic has challenged many aspects of maritime operations worldwide. In addition to the challenges relating to crew changes, maintenance, and surveys, the pandemic has also affected the volume of the merchandise trade across the globe, and influenced the value creation and economic capacity of the industry (UNCTAD 2020). The high person to person rate of the infection has meant that many operations that require physical presence of working personnel now are either suspended or restricted in an attempt to reduce human mobility as protection measures against COVID-19. The cancelation of physical training activities, quarantine, and travel restrictions have created difficulties for the seafarers to acquire or maintain their certificates of competency. The maritime education and training (MET) industry, which forms the platform of skilled manpower supply for the maritime domain, is also facing an unprecedented challenge to ensure the continuity of the MET practices, and to cope with and adapt to the constraints imposed by the COVID-19 pandemic.

Historically, the vocational approach to skill acquisition has been dominant in MET. This approach stresses the importance of “hands on” or practical skills that can be used to carryout everyday operations onboard (Manuel 2017). Such forms of education for maritime trainees meant a fixed time period in the role of apprentice onboard ships, while the skills were transferred and acquired from more experienced peers. In contemporary MET practices, simulator-based training and education are used as complimentary means that can help seafarers acquire some of the pre-requisite competencies for their onboard roles. The use of simulators is regulated by the International Convention on Standards of Training, Certification and Watchkeeping for seafarers (STCW), 1978 as amended, which in certain circumstances, also permits their use as a substitute for onboard training to a limited degree (IMO 2011). The trend for the simulator-based MET has been towards increasing fidelity of the simulators, whereas also focusing on matching appropriate scale and suitability of the simulator to the ever-changing role of the seafarers. The classical definition of the term “fidelity” can be described as the ability of the simulator to closely replicate the real environment, which is central to any discussions regarding simulators (Hays 1980; Kinkade and Wheaton 1972). Technological advancements and cost-effectiveness together with the high focus on safety and sustainability are pushing the availability and possibilities of increased fidelity simulator solutions forward. From the desktop-based simulators that are used for limited replicability of onboard task functions to some of the recent highly realistic full-mission simulators as well as immersive virtual reality simulators (Mallam et al. 2019), simulator-based MET has made significant developments and advancements in recreating realistic working environment at sea. However, the COVID-19 pandemic and consequent restrictions on physical training activities have presented new challenges to the MET institutions. It is high time the technological advancement and innovation in training design are used to make simulator training more accessible to the students using all available media (e.g., desktop, virtual reality, full-mission as well as cloud-based simulators) with or without physical presence of the students and instructors at the simulator training centers.

In this light, the aim of this paper is to review the existing varieties of bridge simulators employed in MET and to analyze how the changes in physical training activities amidst COVID-19 pandemic would affect their evolution in the long term. In order to present a coherent depiction of the continuum of simulator-based MET, we first review four common types of simulators, discuss their characteristics, advantages, and limitations, and further elaborate on how different types of simulators can provide opportunities of novel application in post COVID-19 era. Recommendations for future research explorations are also provided.

## The use of simulators in maritime education and training

Cambridge University Press (2021) termed simulation as “a model of a set of problems or events that can be used to teach someone how to do something, or the process of making such a model”. Simulators, as we know them in present day industrial context, have been in use for many years in safety critical industries such as aviation, process, health care, nuclear, maritime, and rail to prepare the personnel in these domains for their job roles and to ensure that they perform optimally as a team in instances of highly stressful situations (Crichton 2017). Technological advancement has steadily increased the effectiveness of simulators and brought a wide array of advantages to prospective seafarers, as elaborated and summarized in the following Table 1. One of the main advantages is that it provides a non-threatening environment in which trainees are allowed to exercise their skills with the freedom to fail, and to practice their job roles, in presence of instructors and other peers, without any possibility of their errors translating to economic costs, environmental pollution, or in worse cases fatalities (Sharma et al. 2019; Håvold et al. 2015).

**Table 1:** Advantages of using simulators in maritime training and assessment

Dimensions	Advantages of using simulators in maritime training and assessment
Safety	Provide safe environment to practice and rehearse high-risk tasks without dangerous implications
	Allow the trainees to simulate accident scenarios
Flexibility	Allow playback of task performance which enables detailed feedback and discussions
	Simulators are potentially available at all hours which provide time flexibility for designing training programs and facilitate proficiency
	Simulators facilitate the controllability of different scenarios, determining metrological conditions as well as giving the opportunity to the trainees for repeated exercise at their own pace
Skill acquisition	Support transfer of training and skill acquisition
	Allow for tailored delivery of training content
	Enable the trainees to gain understanding regarding their consequences of actions and learn from errors
Skill utilization	Provide opportunities for trainees to apply and utilize the existing and newly acquired knowledge and skills
	Provide opportunities for trainees to practice non-technical skills (e.g., leadership, communication, decision-making skills)
Efficiency	Highly efficient training e.g., trainees can change between any scenario from low speed to high speed, or from narrow channel to open sea navigation without any delay
Cost-effectiveness	Lower operating costs in comparison to on-the-job training
Assessment	Data can be generated through recordings and used to improve training practices and pedagogical approach

Furthermore, the simulation technology facilitates the trainees to learn navigational skills, ships’ reactions, and behaviors in a risk-free environment (Bhaskaran 2018; Hontvedt and Arnseth 2013; Sellberg 2018). It allows playback of task performance which enables detailed feedback and discussions (Bhaskaran 2018) and also offers the possibility for the instructors to tailor the training content, and to monitor and assess the learning outcomes (Maran and Glavin 2003; Sellberg 2018). The use of simulators could also offer the possibility to facilitate trainees to practice non-technical skills (e.g., leadership, communication, decision-making) (Elashkar 2016; Kim and Gausdal 2017; Kim et al. 2021). Section A-I/12 of the STCW code has provided the general guidance regarding the use of maritime simulators, their performance standards, and instructions regarding the training and assessment of competency (IMO 2011).

### Types of simulators

Various simulators exist in the maritime domain, related to the operation they replicate, such as ship bridge simulator, cargo handling simulator, dynamic positioning (DP) simulator, survival craft and rescue boat operations simulator, global maritime distress and safety system (GMDSS) simulator, and vessel traffic service (VTS) simulator (Zghyer and Ostnes 2019). MET institutions around the world have different composition of simulators, depending on several factors such as the simulator provider, training purpose, and resources.

**Fig. 1:**
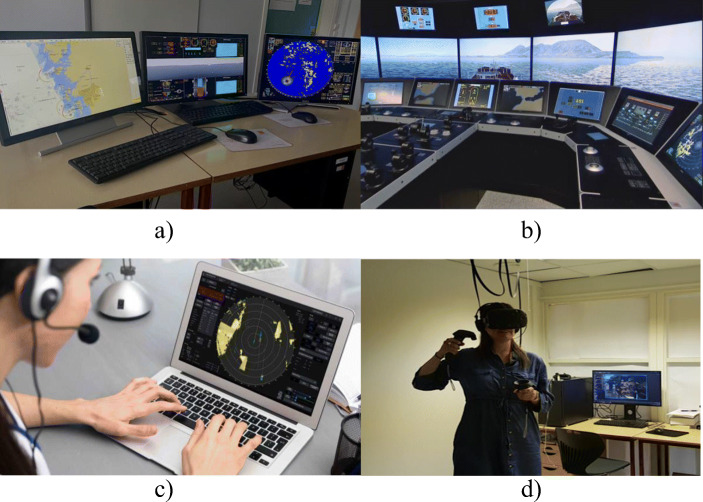
Examples of simulators used in maritime education and training (image credits (c)—Kongsberg Digital AS)

The ship bridge simulators are used to replicate operations conducted on a ship’s navigation bridge, which can be classified into four classes based on their capabilities: class A (full mission); class B (multi-task); class C (limited task); and class S (special task) (DNV 2021). They can also be classified and discussed according to the different technology and hardware used by them. In this study, we elaborate on four types of simulators i.e., desktop-based simulators, full-mission simulators, virtual reality (VR) simulators, and cloud-based simulators (see Fig. 1). Desktop-based simulators consist of desktop computers that replicate some aspects of the maritime operations using ordinary desktop computers that have pre-loaded simulation software. They can be classified as low-fidelity simulators that are able to re-create some aspects of the operational scenarios and enable the trainees to go through them in a limited manner (Wahl and Kongsvik 2018). Full-mission simulators consist of dedicated space in the MET facility that realistically mimic the bridge of a ship with replicas of all essential instrumentations and displays. They are currently the preferred simulator type for addressing most of the regulatory requirements and workplace learning demands due to good level of fidelity offered. However, they are relatively resource intensive to acquire and maintain.

The virtual reality (VR) simulator immerses the users with a realistic experience of the work environment with the use of wearable head-mounted display (HMD). Although the technology with respect to VR was developed back in the 1960s, the usability and popularity of this system were limited until 2010s and it is relatively novel in terms of its application within maritime industry with increasing recognition and investment towards it by the relevant stakeholders (Mallam et al. 2019). Latest development of VR ship bridge simulator could also facilitate multiple users to train and collaborate together in the same training scenario on the same or on different ships while situated around the world. The facility requires moderate monetary investment and could be relatively easy to maintain in comparison to other simulation methods, due to limited workspace required for upkeep and high mobility for the headset and components for transportation either to different MET facilities or on the ship itself.

In contrast to the abovementioned simulator types, cloud-based simulator is one of the latest developments in maritime simulation. The advances in simulation technology and equipment have enabled remote simulation delivery with reduced need for physical presence and increased virtual training activities. Cloud-based simulation enables the instructors and trainees to run the simulation online using a web browser with their own devices (e.g., PCs, laptops, tablets, or mobile devices), which offers the opportunity for the MET institutions and trainees to continue the education and training practices in a standardized manner despite the current challenges posed by COVID-19. However, these features are also some of its possible limitations as the fidelity experienced is dependent on the device used and at the moment such features are only available for desktop, laptop, or tablet interfaces. In addition, the communication undertaken also by extension is not comparable to actual face to face interaction usually done in a MET facility.

### Characteristics of simulators and effects on learning

It is also worth expanding on one of the central characteristics of the simulators as mentioned in the earlier section i.e., the level of fidelity offered by them to the trainees and the instructors. The academic discussion of fidelity in training devices can be traced back to 1970s, with a series of research proposed a variety of fidelity terms and concepts (Hays 1980; Kinkade and Wheaton 1972; Schoenherr and Hamstra 2017). Prior research has synthesized the relevant studies and found that the fidelity term has four dimensions which include physical, functional, behavioral, and perceptual (psychological) fidelity (Hays 1980), depending on the ability of simulators to accurately replicate the technical characteristics, task, and social factors for the targeted operations.

The term physical fidelity pertains to the physical properties of the simulator and the degree to which the simulator could replicate the physical appearance of the actual system (Allen et al. 1986; Kinkade and Wheaton 1972; Liu et al. 2008). Functional fidelity refers to the functional similarity and the degree to which the simulator could replicate the functions and experience of the operational setting in question (Hays 1980). The physical and functional fidelity is also termed together as technical fidelity (Hontvedt and Øvergård 2020). In relation to ship’s bridge operations, this would mean the extent to which the human-machine interfaces (HMIs) of the simulator could closely resemble those of which found on a standard ship bridge, and the extent to which the simulator gives a sense of motion (rolling and pitching) similar to during an average voyage and whether the sounds that are usually present on a ship’s bridge can be replicated accurately. Behavioral fidelity refers to the task similarity, the extent to which the tasks and behaviors required in the simulator setting are similar to the actual tasks in the empirical setting (Hays 1980). The term psychological fidelity, on the other hand, refers to the degree to which the tasks required to be learned by the trainees in the simulator are accurately depicted. In other words, this means that whether the trainees are cognitively and meta-cognitively engaged in problem solving activities within a task on simulator in the same manner as they would in real operational scenario (Hontvedt and Øvergård 2020; Liu et al. 2008). In the context of ship’s bridge operation, this would translate to whether the trainees are performing the same sequence of operations with similar level of stress and workload as they would normally encounter on ship’s bridge. In addition to the physical, functional, behavioral, and perceptual (psychological) fidelity, researchers have also added another dimension i.e., social fidelity, into the discussion. The term “social fidelity” refers to the nature of social interactions as a part of collaborative activities that are embedded into the simulation scenario (Wahl 2020). Hontvedt and Øvergård (2020) used the term “interactional fidelity” to refer to the simulation of team coordination and collaboration in socio-technical environments. In terms of bridge operations, this would mean whether or not the task and operational scenario in the simulator exercise could facilitate interaction between all participants in the simulation and facilitate acquisition of non-technical skills that are required for ship’s bridge operations.

Regarding the effects of simulator fidelity on learning, there have been academic debates across various industrial sectors on whether high-fidelity is superior to low-fidelity simulation (Butler et al. 2009; Massoth et al. 2019; Schoenherr and Hamstra 2017). It has been well acknowledged that increasing the fidelity of simulators would not necessarily improve learning outcome. The relationship between learning effect and fidelity is non-linear (Rieber 1994). Although there are studies showing that high-fidelity simulators could lead to better training experience with enhanced practice and learning (Hoadley 2009), related research has also suggested that the use of high-fidelity simulators does not necessarily maximize the learning outcome, as competence development do not rely solely on highly context-specific environments (Dahlstrom et al. 2009). Low-fidelity simulators simplify the visual and environmental aspects of real work environment, whereas high-fidelity simulators render visual as well as auditory and motion cues in lifelike simulations (Sellberg 2017). However, low-fidelity simulators also have the potentials to provide greater pedagogical and economic advantages. There is also evidence regarding effectiveness of low-fidelity simulators when training for relatively simple cognitive tasks and procedures (Maran and Glavin 2003). Fidelity has limited implications beyond the trainee’s heightened motivation (Jacobs 1975), and may lead to cognitive biases (e.g., overconfidence issues) and diagnostic errors (Massoth et al. 2019). Nevertheless, Hontvedt (2015) argued that the degree of fidelity in simulator training must match the requirement of the work tasks and learning objectives. In the context of learning efficiency, low-fidelity simulators are considered effective for novice trainees before moving into the high-fidelity simulators for more complex tasks (Alessi 1988). On the other hand, in terms of acquisition of non-technical skills, Lewis et al. (2012) have found that the use of high-fidelity simulator associates with improved interpersonal communications skills, team behavior, and leadership skills and aids in developing trainee’s self-efficacy and confidence in their competence. In addition, Sellberg (2017) also pointed out the importance of coordinated representation of real workplace through talks and gestures during simulator exercises to prevent negative skill transfer caused by lack of realism thereby shifting the focus to the quality of instructions rather than simulator fidelity during exercises.

## Analysis methodology

To comprehensively analyze the four common types of bridge simulators and to analyze how the changes in physical training activities amidst COVID-19 pandemic would affect the evolution in the long term, a strengths, weaknesses, opportunities, and threats (SWOT) analysis was performed in this study through focus group discussions as well as related documents and research results. SWOT analysis is a common and widely recognized method for identification and assessment of the strengths, weaknesses, opportunities, and threats related to a system or activity, and the results could provide a basis for strategy formulation and decision-making (Gürel and Tat 2017). The strengths and weaknesses refer to the internal factors related to the organization, activity, or product in question, whereas the opportunities and threats refer to the external factors affecting them (Helms and Nixon 2010). In the context of the present study, two focus group discussion workshops were organized in August and October 2020 with seven maritime researchers in collaboration for brainstorming and SWOT analysis, and the researchers can also be considered subject matter experts (SMEs) who engage heavily in simulator-based MET at the affiliating institution where three levels (i.e., bachelor, master, and PhD level) of maritime education and training are provided. The focus group discussion workshops were the part of initiatives taken by the institute regarding the efforts to counter the expected uncertainty regarding ongoing COVID-19 pandemic and optimal utilization of the simulator resources available for maritime simulator-based training and assessment. The workshops consisted of iterative ideation and brainstorming sessions followed by synthesis and evaluation of each point contained in the analysis. The focus of the study was on ship’s bridge operations and consequently all the discussions hovered around the various types of simulators available for simulating the bridge operations. The notetaking and documentation were maintained which was studied in detail subsequent to the workshop session.

## SWOT analysis and discussions

The SWOT analysis for the different types of simulators is based on the internal factors such as their fidelity, scalability, and accessibility, the characteristics which are inherent to them and can act as strength or weakness depending on the context of use. Whereas the external factors are considered to be impact of COVID-19 and technological advancements, the same can present various opportunities and threats for their utilization and viability. The SWOT analysis results are presented in the following Table 2.

**Table 2:** Maritime simulator SWOT analysis

Type of simulator	Internal factors		External factors	
	Strengths	Weaknesses	Opportunities	Threats
Desktop (DT) simulators	DT.S1 - Used for product/equipment familiarization DT.S2 - Small learning curve for both instructors and students DT.S3 - Ease of access and setup for students and instructors DT.S4 - Instructor could give formative assessment DT.S5 - Adjust the learning pace and complexity of the learning during the exercise DT.S6 - Multipurpose	DT.W1 - Lack of fidelity DT.W2 - Need dedicated infrastructure and space DT.W3 - Lack of training for team cooperation DT.W4 - Lack of immersivity	DT.O1 - Potential for new roles such as shore control center simulation DT.O2 - Use in geographically separated synchronous learning DT.O3 - Adaptive to future training needs DT.O4 - Easy to procure and scalable compare to full-mission simulators	DT.T1 - Irrelevance or replacement by other simulators DT.T2 - Gamification DT.T3 - Difficulty in utilization during COVID-19
Full-mission (FM) simulators	FM.S1 - High physical and social fidelity FM.S2 - Appropriate for all STCW training requirements FM.S3 - Beneficial for NTS development and team training FM.S4 - Possibility to observe the dynamics of the bridge room FM.S5 - Physical interaction with the bridge team	FM.W1 - Costly to acquire and maintain FM.W2 - Need highly competent instructors FM.W3 - Require frequent and resource-intensive maintenance FM.W4 - Takes time to develop, adjust, and improve exercise content and quality	FM.O1 - Utilization with combination of new technologies—VR/AR FM.O2 - Combination with other means of data collection—eye tracking, heart rate variability, etc. for assessment	FM.T1 - Difficulty in utilization during COVID-19 FM.T2 - Replacement due to economic unviability
VR simulators (VR)	VR.S1 - Highly immersive VR.S2 - Supports motivation and higher order learning VR.S3 - High mobility VR.S4 - Innovative and attractive training solution VR.S5 - Real-time feedback from trainees (AR)	VR.W1 - Motion sickness, depth perception issues VR.W2 - Steep learning curve VR.W3 - Learning effect is questionable VR.W4 - Lack of student-instructor interaction VR.W5 - Limited team cooperation and interaction	VR.O1 - Low-cost training per head VR.O2 - Remote/onboard training VR.O3 - Fast paced research and development in VR	VR.T1 - Less utilization due to lack of universal design VR.T2 - Physical distance constraints VR.T3 - Lack of training scenarios VR.T4 - Danger of technology hype VR.T5 - Technological acceptance barriers
Cloud-based (CB) simulator	CB.S1 - Ubiquitous learning CB.S2 – Self-directed CB.S3 - Less capital intensive CB.S4 - Limited maintenance of hardware CB.S5 - No need of physical presence	CB.W1 - Lack of social interaction CB.W2 - Lack of formative assessment CB.W3 - Limited transfer of learning, unclear application in MET CB.W4 - Lack of team training opportunities	CB.O1 - Geographically separated synchronous learning CB.O2 - Novel mode of training and assessment CB.O3 - Possible use in post COVID-19 era CB.O4 - Highly scalable	CB.T1 - Cyber security CB.T2 - Lack of institutional support CB.T3 - Internet connectivity and speed barriers

In the case of desktop-based simulators (DT), some of the evident strengths are relatively small learning curve (DT.S2), ease of setup (DT.S3), and potential of their multipurpose use (DT.S6). However, their lack of fidelity (DT.W1), ability to be employed for team training scenarios (DT.W3), and immersivity (DT.W4) limits their utilization for greater number of operations. The desktop simulators still present opportunities for their use in new roles (DT.O1) and in geographically separated synchronous learning (DT.O2) among other benefits. Fast pace of technological developments and by extension their irrelevance in near future (DT.T1) in addition to the difficulty in imposing infection control measures due to their size and design (DT.T3) can present some of the threats which could lead to them being overlooked for most of the functions in favor of other types of simulators.

The full-mission (FM) simulator setup in contrast provides high level of fidelity (FM.S1) and is indispensable for training with regard to non-technical skills (FM.S3). High investments in terms of setup costs, maintenance (FM.W1,3), complexity in planning and executing exercise scenarios (FM.W4), and the need for skilled instructors for their optimal use (FM.W2) can be argued to be some of their limitations. Despite these, full-mission simulators will continue to play dominant role owing to their versatility and fidelity. They can be used in conjugation with other emerging simulator technologies such as VR (FM.O1) and can also accommodate new methods of performance data collection such as eye tracking (FM.O2). The limitations imposed by the need for adequate social distancing pose a threat to their role and use within MET as uncertainty regarding COVID-19 pandemic duration continues (FM.T1). One common constraint in the case of both DT and FM simulators is their limited ability to support remote learning which becomes the focus in ongoing MET developments and discussions. In contrast to this, the virtual reality (VR) simulators or the cloud-based (CB) simulators have the potential to be employed to deliver distributed learning solutions for the MET community specially in the post COVID-19 era.

The VR simulators are highly immersive (VR.S1), mobile (VR.S3), and innovative (VR.S4) in terms of their application to MET and can transform the training maritime training and assessment practices. However, motion sickness and eye fatigue issues (VR.W1) along with steep learning curves for both trainees and instructors (VR.W2) are pointed out as some of the core weaknesses of VR simulators. Although several studies have reported statistically significant improvement in task performance or technical skills after VR simulator training (Aïm et al. 2016; Haque and Srinivasan 2006), the motivation and engagement among maritime trainees and the effect of learning still need further explorations (VR.W3). Notwithstanding such issues, VR simulators, owing their high mobility, provide opportunities for the trainees to be trained from any location, even from the ships itself (VR.O2). After initial investments into the simulators, long-term dividends, in terms of low-cost training per head (VR.O1), make them highly feasible in post COVID-19 applications. The questions surrounding their usage in terms of design issues (VR.T1) and infection control measures (VR.T2) albeit can limit their utilization if not addressed sufficiently. The CB simulators have the potential to make ubiquitous simulations in MET a reality (CB.S1) and this along with relatively less requirements of resources (CB.S3,4) is their biggest advantages. However, they can severely limit the interaction between trainees and instructors with no opportunity for face-to-face contact. This characteristic, though advantageous in post COVID-19 application, is also its limitation (CB.W1,4). While they can enable geographically separated synchronous learning (CB.O1) and allow for novel modes for training and assessment in such situations (CB.O2), they can potentially be vulnerable to cyber-attacks that can target main databases (CB.T1). Low internet speed and connectivity issues in some geographical areas (CB.T3) could further limit their utilization and effectiveness on learning. A common concern surrounding both VR and CB simulators is their early phase in technological maturity. It could take certain time period before they can be properly inculcated in their use for different scenarios and operational application for MET.

As described above, there are variety of simulator types available for maritime education and training practices, with each offering certain advantages and limitations depending on the context of the use. Desktop-based simulators can be thought of the first-generation simulators with relatively low investment and maintenance costs. Despite several years since their introduction, they still have considerable use in MET facilities, specially, for the initial training and familiarization purposes. They offer the possibility to scale and adapt them for novel maritime operational roles. The only concerns regarding their use are their potential obsoleteness with further advance in simulator technologies and availability of ubiquitous learning solutions in tablets and mobile devices. At present, the full-mission simulators are considered to be most versatile in use and which best supports the MET facilities in meeting the regulatory requirements and training objectives. Their replicability of operational experience and the ability to train both technical and non-technical skills for the trainees in a highly controlled and quasi-real environment is unmatched at the moment. High investments and maintenance costs along with mobility and usability issues, especially in phase of COVID-19 pandemic, are some of its limitations. However, full-mission simulators have the potential for further use in combination with advances in VR, as well as cloud-based technologies and can be used for further innovative pedagogical applications. They require highly competent and trained instructors in their usage and investments also in terms of time when designing exercise scenarios and learning activities in their platform. The desktop-based and full-mission simulators represent the dominant simulator types in use currently at MET facilities. The studies regarding their utilization, interaction issues, fidelity experienced, and design of training and assessment interventions can be found in MET research literature. In contrast to these two simulator types, the VR simulators and cloud-based simulators are gaining their significance and popularity in transforming how MET is delivered.

In the case of VR simulators, authentic learning experience can be offered to the trainees because of the high immersivity. It is possible for the students to better articulate their understanding by interacting with the virtual objects. VR-based simulators, therefore, directly support two of the learning theories, namely experiential learning and constructivism (Pantelidis 1995). Research literature suggests that VR can support learner motivation and engagement and promote higher order learning (Di Natale et al. 2020; Mallam et al. 2019). It is also possible to get overall greater return on training investments, as the VR simulators can be used in different settings due to their compactness and high mobility. However, it is worth noting that learning community has also recognized some of the limitations with VR. There is an initial learning curve related to the use of new technology itself which might require the instructors to invest considerable amount of time and efforts to put them into practice (Aldunate and Nussbaum 2013). In addition, motion sickness, eye fatigue, and lack of direct feedback during the tasks are some of the evident issues that are arising with increased use (Munafo et al. 2017). There are also concerns regarding the technology “hype” around VR and it is argued that it might require certain time period before the actual benefits and limitations with respect to its use are properly established (Cochrane and Farley 2017). Despite certain limitations, VR-based simulators continue to be promising development in the case of maritime simulation due to potential novel application for training and educational purposes. Similar expectations are towards the latest simulator types being introduced for maritime training and educational purposes i.e., cloud-based simulators. CB simulators have the potential to introduce ubiquitous learning solutions that could enable large-scale distributed learning communities in maritime industry. With minimum support and maintenance required for its upkeep and operation, it can also reduce training costs per head for the MET facility. The enabling of remote learning delivered at personal devices used by the trainees and the possibility of learners participating together from any geographical location is a leap forward from the existing simulator solutions. However, these features are also some of the possible limitations of cloud-based simulators. With limited means of interaction and no actual presence, the fidelity experienced as an extension is relatively less compared to other simulator types. Also, the means of providing feedback to the students and contributing in their formative assessment is limited. At the end of this discussion, it is worth noting that the development of VR and CB simulators for the bridge operations are at early stages, and their affordances and capabilities in coming years might differ from what assumed in this paper.

## Simulator-based MET in the post COVID-19 era

The adoption of new digital modes of learning solutions could not only be used to minimize physical presence and contact between trainees and instructors under the current COVID-19 situation, but also lead to introduction of novel modes of maritime training and assessment. The technological changes, if implemented correctly, have dual potential of reducing the risk of transmission of disease, as well as savings in terms of time and resources for the MET institutions, employers, and seafarers.

Among the 17 Sustainable Development Goals (SDG) established by the United Nations, SDG 4 is aimed to “ensure inclusive and equitable quality education and promote lifelong learning opportunities for all” (UN, 2020). Ensuring that a greater number of people receive the required vocational and technical skills is one of the ways SDG 4 can be achieved (WMU 2019). In this context, digital and remote learning opportunities through CB and VR simulators have the potential to provide the paradigm shift required for MET in post COVID-19 era, as presented in the previous section. Maritime institutions, instructors, and companies need to search for ways to repair the damage caused by COVID-19’s interruptions to learning trajectories. In addition, changing the mediums for instructional delivery requires several stakeholders to rethink post-pandemic pedagogical approach. It could be necessary to apply a combination of face-to-face, blended, and online learning methods as well as revision of the existing curriculum to achieve these objectives.

Pedagogical utilization of simulators comes through proper selection of simulators and effective assessment techniques both of which rely heavily on the efficiency of simulator instructors (Tsoukalas et al. 2008). As Sellberg (2018) notes, the simulator technology itself does not teach the specifics of the job and the sequence in which tasks are to be executed by the students. The learning occurs through the active role played by the trainees in the simulation session and supported by the instructors in orienting the trainees and scaffolding throughout the process. Similarly, Wahl (2020) demonstrated that the learning occurring in the maritime simulation-based training and education is not solely influenced by the physical and psychological fidelity. She commented on the importance of social fidelity i.e., the extent to which the trainee-instructor interactions in the simulator and the joint collaboration activities exercised as if in the actual work environment. Hontvedt and Arnseth (2013) demonstrated the importance of structuring the role play and promoting interactions in maritime simulator settings. They utilize socio-cultural theoretical lens and interaction analysis to understand how learning takes place in these situations and the significance of establishing relevant context of training activities. Wahl and Kongsvik (2018) underlined the need for context-specific training of behavioral markers during social interactions in the classroom-based training as well as in the simulators. Sellberg (2018) also argues that the interaction and guidance that occurs in action is an important contributor in reaching the student’s learning goals.

With novel remote access simulators, such as cloud-based simulations, new practices must be developed to substitute these well-established pedagogical methods. The challenges of ensuring that students reach the intended learning goals when they run simulations, without active instructor support, have been researched in other fields but need further research in maritime simulators. With the introduction and adaptation of new types of simulators, namely VR and CB simulators, the need for addressing the training and assessment practices will require renewed focus. As elaborated above, these simulator types offer increased fidelity and interaction experiences than those offered by conventional desktop and full-mission simulators. However, there is a lack of evidence regarding their training outcomes, since most non-technical training approaches are focused on high-fidelity simulation without thoroughly defined training goals (Praetorius et al. 2020). Therefore, the effect of simulator types and levels of fidelity on acquisition of both technical and non-technical skills for maritime trainees need comparison and evaluation. This could provide evidence regarding their suitability of use in particular contexts with optimum allocation of resources for MET facilities depending on the training objectives. Furthermore, with the possibility of mass scale participation remotely and an element of role play in virtual environments, it will be interesting to design and analyze self-directed, collaborative training scenarios that are hypothesized to support acquisition of necessary skills in generic learners (Lee et al. 2014). These developments can also have implications for some of the new operational roles, as well as increasing digital distributed learning practices that are only accelerated as a response to COVID-19.

## Summary and conclusion

In summary, the following steps can be taken by the stakeholders in MET to meet the existing challenges as well as for charting out the future course of action in relation to the advancements of simulator-based learning in MET:

1. Effective utilization of both quantitative and qualitative research for comparing and evaluating the effectiveness of different types of simulators, particularly, VR and CB simulators for their application in variety of operational scenarios.
2. Revision of contents in the simulator-based MET curriculum as well as of delivery methods owing to the increased use of distance learning tools during COVID-19 pandemic.
3. Focus on self-directed learning, collaborative interaction, and scaffolding methods that are suitable for distance learning as well as for the future skillsets required in maritime domain.
4. Support towards MET instructors through competence development and resources allocation to adequately deal with changes required in maritime training and assessment.

Changes in the educational practices due to technological advancements and constraints brought by COVID-19 are forcing the maritime industry in exploring the viability and effectiveness of different digital distributed learning solutions and the review of practices in the existing maritime simulator-based training and education. However, the instructors involved in MET institutes will continue to play a key role in ensuring that technology integration to support achievement of learning outcomes can be carried out seamlessly and in cultivation of digital community of learners. One of the most defining characteristics of the cloud-based simulators as well as virtual reality simulators is their enabling of large-scale distributed modes of learning, which gives the potential to introduce novel training and assessment practices within MET and promote wide access and quality education for the seafarers, thereby contributing to the SDG 4 initiatives of the United Nations. Future research should be directed in exploring, validating, and enhancing the effectiveness of cloud-based simulators as well as virtual reality simulators in maritime domain.

## References

[CR1] Aïm F, Lonjon G, Hannouche D, Nizard R (2016). Effectiveness of virtual reality training in orthopaedic surgery. Arthroscopy: The Journal of Arthroscopic & Related Surgery.

[CR2] Aldunate R, Nussbaum M (2013). Teacher adoption of technology. Comput Hum Behav.

[CR3] Alessi SM (1988 Fidelity in the design of instructional simulations. Journal of computer-based instruction

[CR4] Allen JA, Hays RT, Buffardi LC (1986). Maintenance training simulator fidelity and individual differences in transfer of training. Hum Factors.

[CR5] Bhaskaran. (2018) Importance of simulators in maritime training. International Journal of Research and Analytical Reviews*, 5*(4)

[CR6] Butler KW, Veltre DE, Brady D (2009). Implementation of active learning pedagogy comparing low-fidelity simulation versus high-fidelity simulation in pediatric nursing education. Clinical Simulation in Nursing.

[CR7] Cochrane T, Farley H (2017) Special issue on mobile AR & VR: integrating SOTEL in learning design. Australas J Educ Technol*,* 33(6)

[CR8] Crichton MT (2017). From cockpit to operating theatre to drilling rig floor: five principles for improving safety using simulator-based exercises to enhance team cognition. Cogn Tech Work.

[CR9] Dahlstrom N, Dekker S, van Winsen R, Nyce J (2009). Fidelity and validity of simulator training. Theor Issues Ergon Sci.

[CR10] Di Natale AF, Repetto C, Riva G, Villani D (2020). Immersive virtual reality in K-12 and higher education: a 10-year systematic review of empirical research. Br J Educ Technol.

[CR11] DNV (2021) Maritime simulator systems. https://rules.dnvgl.com/docs/pdf/DNVGL/ST/2017-03/DNVGL-ST-0033.pdf

[CR12] Elashkar MA (2016). The use of simulation techniques in the development of non-technical skills for marine officers. International Journal of General Engineering and Technology (IJGET).

[CR13] Gürel E, Tat M (2017) SWOT analysis: a theoretical review. J Int Soc Res. 10(51)

[CR14] Haque S, Srinivasan S (2006). A meta-analysis of the training effectiveness of virtual reality surgical simulators. IEEE Trans Inf Technol Biomed.

[CR15] Håvold JI, Nistad S, Skiri A, Ødegård A (2015). The human factor and simulator training for offshore anchor handling operators. Saf Sci.

[CR16] Hays RT (1980) Simulator fidelity: a concept paper. US Army Research Institute for the Behavioral and Social Sciences

[CR17] Helms MM, Nixon J (2010) Exploring SWOT analysis–where are we now? A review of academic research from the last decade. Journal of strategy and management

[CR18] Hoadley TA (2009). Learning advanced cardiac life support: a comparison study of the effects of low-and high-fidelity simulation. Nurs Educ Perspect.

[CR19] Hontvedt M (2015). Professional vision in simulated environments—examining professional maritime pilots’ performance of work tasks in a full-mission ship simulator. Learn Cult Soc Interact.

[CR20] Hontvedt M, Arnseth HC (2013). On the bridge to learn: analysing the social organization of nautical instruction in a ship simulator. Int J Comput-Support Collab Learn.

[CR21] Hontvedt M, Øvergård KI (2020). Simulations at work—a framework for configuring simulation fidelity with training objectives. Computer Supported Cooperative Work (CSCW).

[CR22] IMO (2011). International Convention on Standards of Training, Certification and Watchkeeping for Seafarers,(STCW) 1978, as amended in 1995/2010.

[CR23] Jacobs RS (1975) Simulator motion as a factor in flight simulator training effectiveness 10.1177/001872087301500608 4783187

[CR24] Kim T-E, Gausdal AH (2017). Leading for safety: a weighted safety leadership model in shipping. Reliab Eng Syst Saf.

[CR25] Kim T-e, Sydnes AK, Batalden B-M (2021). Development and validation of a safety leadership Self-Efficacy Scale (SLSES) in maritime context. Saf Sci.

[CR26] Kinkade RG, Wheaton GR (1972) Training device design. Human engineering guide to equipment design:668–699

[CR27] Lee K, Tsai PS, Chai CS, Koh JHL (2014). Students’ perceptions of self-directed learning and collaborative learning with and without technology. J Comput Assist Learn.

[CR28] Lewis R, Strachan A, Smith MM (2012). Is high fidelity simulation the most effective method for the development of non-technical skills in nursing? A review of the current evidence. The open nursing journal.

[CR29] Liu D, Macchiarella ND, Vincenzi DA (2008) Simulation fidelity. Human factors in simulation and training:61–73

[CR30] Mallam SC, Nazir S, Renganayagalu SK (2019). Rethinking maritime education, training, and operations in the digital era: applications for emerging immersive technologies. J Mar Sci Eng..

[CR31] Manuel ME (2017). Vocational and academic approaches to maritime education and training (MET): trends, challenges and opportunities. WMU J Marit Aff.

[CR32] Maran NJ, Glavin RJ (2003). Low-to high-fidelity simulation–a continuum of medical education?. Med Educ.

[CR33] Massoth C, Röder H, Ohlenburg H, Hessler M, Zarbock A, Pöpping DM, Wenk M (2019) High-fidelity is not superior to low-fidelity simulation but leads to overconfidence in medical students. 29BMC Med Educ. 19(1) 10.1186/s12909-019-1464-7 PMC6341720 30665397

[CR34] Munafo J, Diedrick M, Stoffregen TA 2017. The virtual reality head-mounted display Oculus Rift induces motion sickness and is sexist in itseffects. Experimental brain research, 235(3), 889-901. 10.1007/s00221-016-4846-7 27915367

[CR35] Pantelidis VS (1995). Reasons to use virtual reality in education. VR in the Schools.

[CR36] Praetorius G, Hult C, Österman C (2020). Maritime resource management: current training approaches and potential improvements. TransNav, International Journal on Marine Navigation and Safety of Sea Transportation.

[CR37] Press CU (2021) Simulation. In Cambridge Dictionary. Retrieved April 20, 2021, from https://dictionary.cambridge.org/dictionary/english/simulation

[CR38] Rieber LP (1994) An instructional design philosophy of interaction based on a blending of microworlds, simulations, and games

[CR39] Schoenherr JR, Hamstra SJ (2017). Beyond fidelity: deconstructing the seductive simplicity of fidelity in simulator-based education in the health care professions. Simul Healthc.

[CR40] Sellberg C (2017). Representing and enacting movement: the body as an instructional resource in a simulator-based environment. Educ Inf Technol.

[CR41] Sellberg C (2018). From briefing, through scenario, to debriefing: the maritime instructor’s work during simulator-based training. Cogn Tech Work.

[CR42] Sharma A, Nazir S, Ernstsen J (2019) Situation awareness information requirements for maritime navigation: A goal directed task analysis. Safety Science 120:745-752

[CR43] Tsoukalas VD, Papachristos DA, Tsoumas NK, Mattheu EC (2008). Marine engineers’ training: educational assessment for an engine room simulator. WMU J Marit Aff.

[CR44] UNCTAD (2020) Review of maritime transport 2020. Retrieved from https://unctad.org/en/PublicationsLibrary/rmt2019_en.pdf

[CR45] Wahl AM (2020). Expanding the concept of simulator fidelity: the use of technology and collaborative activities in training maritime officers. Cogn Tech Work.

[CR46] Wahl AM, Kongsvik T (2018). Crew resource management training in the maritime industry: a literature review. WMU J Marit Aff.

[CR47] WMU (2019) Transport 2040: automation, technology, employment-the future of work (Vol. (Online) 978-91-984865-2-0). World Maritime University

[CR48] Zghyer R, Ostnes R (2019) Opportunities and challenges in using ship-bridge simulators in maritime research

